# Abnormal Fixational Eye Movements in Ophthalmic Disease: Implications for Visual Function and Clinical Assessment

**DOI:** 10.3390/jemr19050100

**Published:** 2026-09-09

**Authors:** Sophia Castro, Nancy O. Mahfouz, Simrat Renu, Kathiana Jeanty, Elana Safonova, Doris Prela, Ola Abozid, Ayesha Mulla, Josey M. Spiers, Robert G. Alexander

**Affiliations:** 1 CUNY School of Medicine, New York, NY 10031, USA; 2 New York Institute of Technology, Old Westbury, NY 11568, USA; 3 New York Institute of Technology, New York, NY 10023, USA

**Keywords:** fixational stability, microsaccades, ocular drift, preferred retinal locus, visual acuity

## Abstract

Ophthalmic disorders cause pathological abnormalities in fixational movements. Across disorders, recent studies demonstrate that fixation can be disrupted, often with reported changes in stability and other oculomotor dynamics as the conditions progress. Some of these changes are functionally linked to reduced visual acuity, degraded reading performance, and impaired binocular function. In this narrative review, we synthesize recent work on fixational eye movements and fixation instability in ophthalmic disease. We argue that fixational measures may provide research biomarkers and clinical correlates of disease progression. These metrics could eventually contribute to treatment monitoring applications. The development of accessible and non-invasive eye-tracking tools could enhance clinical interventions. Continued progress will depend on establishing robust normative datasets and determining which oculomotor metrics provide reliable clinical information across disease states.

## 1. Introduction

Eye movements shape what we see and how we see it; conversely, our visual perception can also change the way we move our eyes. In normal vision, even small eye movements allow gaze to be precisely directed to relevant information [1,2], enhance visual processing [2,3,4], and prevent image fading [5,6,7,8]. Even when we feel like we are holding our gaze still, our eyes are in constant motion, producing fixational eye movements such as tremor, drift, and microsaccades. Microsaccades are small saccadic shifts; drift is a slower intersaccadic motion; and tremor consists of very small high-frequency oscillations with amplitudes that approximate the width of a single photoreceptor [9,10]. Of those movements, microsaccades have received the greatest attention in both basic and clinical research, where tremor is rarely assessed due to the technical difficulties involved in measuring such small motions [6].

Ophthalmic disease can produce specific fixational eye movement patterns with distinct characteristics, and objectively assessing these small eye movements aids our understanding of pathologies that impair fixation. Abnormalities in fixational eye movement dynamics and in fixation stability have been linked to reduced visual acuity, degraded reading performance, impaired stereopsis, and other functionally meaningful deficits across multiple disorders. If they demonstrate adequate reliability, validity, and clinical utility, leveraging non-invasive oculomotor metrics may improve assessment and monitoring in patient care [11]. In the long term, they may eventually contribute to refining early and differential diagnostics. Understanding the relationship between eye movements and disease progression may reveal the nature and extent of visual impairments. Abnormalities in eye movements can be detrimental, adaptive or compensatory (and thus improve visual processing by supporting residual visual function). They can also be merely associated with—but not directly related to—impairment. Identifying those relationships can clarify the underlying mechanisms of those abnormalities. If future studies demonstrate sufficient clinical utility and specificity, longitudinal gaze measures may also help evaluate the effectiveness of ongoing treatment. Progression and treatment response could theoretically be evaluated in terms of the normalization of fixational eye movement dynamics, though whether such changes are clinically meaningful remains to be established.

Recent research efforts have aimed to characterize fixation stability and fixational eye movement dynamics in an increasing number of ophthalmological conditions, which we discuss in this review. Despite rapid growth in this area of inquiry, relatively few studies address fixational eye movement impairments in patient populations. In this narrative review, we provide an overview of the literature describing these impairments, with a particular focus on studies that have been published in the past five years. Building on an earlier review in this area [12], the present article provides an updated overview of the substantially expanded literature in this field. We focus on how different disorders alter core oculomotor features and on the extent to which these changes are known to impact visual function. The length of each section below reflects the maturity of the literature. As such, this paper gives relatively greater emphasis to amblyopia, strabismus, and macular disease, for which there are currently the largest and most methodologically developed evidence bases.

### Gaze Fixation Measures

Several terms in this review refer to different levels of measurement and should not be treated as interchangeable. Microsaccades are brief, small-amplitude saccades that shift gaze during fixation; they can be quantified by rate, amplitude, direction, velocity, and timing. See refs. [6,13] for an extended discussion of microsaccades. Ocular drift refers to the slower motion of the eye between saccadic events and is typically described by velocity, dispersion, or spectral properties. Nystagmus refers to rhythmic involuntary oscillations that may contain slow and fast phases, whereas saccadic intrusions are saccadic events that disrupt intended fixation.

General fixation instability is different from these measures. It is a descriptive construct indicating that gaze position is less stable than expected, but—critically—it does not identify the oculomotor source of that instability. Fixation instability is measured in terms of the spatial dispersion of gaze samples. For example, bivariate contour ellipse area (BCEA) is an aggregate spatial-dispersion metric used to assess fixation stability. BCEA estimates the area containing a specified proportion of fixation samples (commonly 68%, 95%, or other predefined contour levels). Larger BCEA values indicate more dispersed gaze, but BCEA does not determine whether that dispersion was produced by drift, microsaccades, other eye movements, calibration error, data loss, or some combination of these and other factors. BCEA and related dispersion measures depend on many factors. These include recording duration, fixation target properties, viewing condition, monocular versus binocular testing, sampling rate, calibration quality, and preprocessing decisions. Results obtained with different devices, tasks, durations, or metrics should not be assumed to be directly comparable. Studies often differ in hardware, sampling rate, calibration, viewing distance, targets, exclusion rules, preprocessing, event detection, and operational definitions. Lower sampling frequency can reduce sensitivity to small or brief movements; calibration error can inflate apparent dispersion; longer recordings can decrease fixation stability; and algorithmic differences can change whether or not the same event is classified as a microsaccade. Despite efforts to establish standardized reporting for eye movement research [14], it is often the case that some relevant factors go unreported. In the absence of standardized acquisition and reporting, apparent disease effects may reflect differences in characteristics unrelated to clinically meaningful oculomotor abnormalities. That being said, this review aims to describe any broadly consistent findings that may suggest the existence of meaningful abnormalities.

## 2. Methods

A narrative review was selected because the relevant literature spans distinct disorders, devices, recording conditions, and outcome definitions. Under these conditions, a single pooled assessment would risk treating non-equivalent measures as though they assessed the same construct and different patient populations, misrepresenting them as homogenous. The stated five-year emphasis was used to identify recent developments but was not an exclusion criterion.

Literature was identified through searches of PubMed, Google Scholar, and Web of Science using combinations of terms. Database-specific search strings, adapted to each platform, included combinations of the following terms: “fixation stability”, “microsaccades”, “ocular drift”, “fixational eye movements”, “ophthalmology”, “myopia”, “amblyopia”, “strabismus”, “glaucoma”, “macular degeneration”, “central scotoma”, “diabetic retinopathy”, “retinal detachment”, and “cataract”. The final search was conducted on 29 April 2025.

We included empirical, methodological, and clinically relevant conceptual studies when topically relevant. We retained foundational studies when they introduced key measures, enabled interpretation of later work, or provided rare longitudinal or treatment evidence. We excluded studies that did not address fixational eye movements or fixation stability, clinically relevant oculomotor outcomes, ophthalmic disease, or English-language evidence. Eligible study types included observational studies, experimental studies, clinical treatment studies, methodological papers, and prior reviews used to establish context.

Records were screened for relevance by the author team through title, abstract, and full-text review as appropriate. Each disease section was drafted from the studies judged most relevant to the specific disorder, measurement approach, and visual-function outcome under consideration.

See Table 1 for an overview of the included studies.

## 3. Myopia and Other Refractive Disorders

While large saccades may not be impacted by myopia [15,47], studies have found abnormalities in fixation stability. In patients with myopia, fixation stability is negatively correlated with axial length (the distance from the front of the cornea to the back of the eye): the greater the axial length, and thus the more myopic, the less stable fixation becomes [15,16,17]. This pattern may reflect differences in microsaccade production: myopia increases the size of microsaccades relative to health controls but does not alter the velocity of the movements or the rate at which they are produced [18]. Microsaccade size also increases with the severity of the uncorrected refractive error. A likely explanation is that precise control of microsaccadic amplitudes may require precise visual information on the fovea [18]. However, this explanation cannot be confirmed from fixation stability measurements alone.

**Figure 1:**
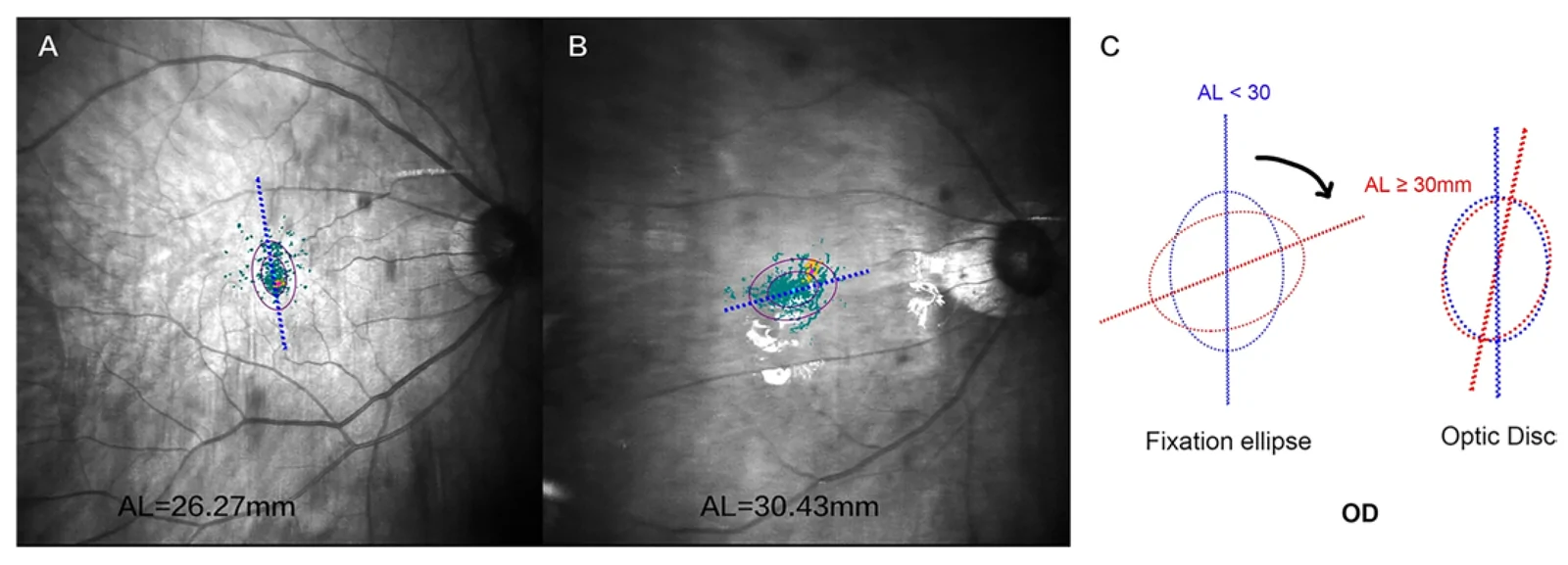
The images show the direction of 63% and 95% BCEA fixation ellipses for patients with different axial lengths (AL). (**A**) The right eye of a 58-year-old male (AL: 26.2 mm) exhibiting a fixation ellipse angle of 97.4°. (**B**) The right eye of a 50-year-old male (AL: 30.43 mm) demonstrating a more horizontal fixational ellipse angle of 13°. (**C**) The fixation ellipse undergoes progressive superior rotation as AL increases, with the magnitude of this rotation exceeding that of the optic disc. Reprinted from Zhu, He [19] without modification, under the terms of the Creative Commons Attribution 4.0 International License (CC BY 4.0).

Zhu, He [19] investigated the effects of myopia on fixation stability. Fixation stability is often measured as a function of gaze dispersion (e.g., the bivariate contour ellipse area [BCEA]), a measure that can be affected by changes in microsaccades or drift but which does not differentiate between the two. See Figure 1 for a visualization of BCEA. Larger BCEA values reflect a more dispersed and thus less stable gaze. BCEA can also vary in orientation, revealing directional biases in gaze behavior. Zhu, He [19] found that in highly myopic patients, axial length is positively associated with superiorly shifted BCEA ellipses. Although the gaze was relatively unstable for the patients, the direction of fixational eye movements is biased such that the visual axis is maintained within the pupil boundary.

## 4. Amblyopia and Strabismus

Uncorrected refractive error (and other conditions) in early childhood can potentially lead to later problems. Uncorrected error can interfere with focusing the eyes, leading to strabismus—a misalignment during fixation. The error can also lead to the brain favoring one eye over the other and the underdevelopment of the visual pathways to the weaker eye, a condition called amblyopia.

Eye tracking measurements in amblyopia and strabismus often reveal reduced fixation stability relative to healthy controls [48,49,50]. This is especially true for patients with large-angle strabismus [51]. This fixation instability results in part from increased drift, fixational saccades that are more disconjugate than those of control participants [20,52], the presence of nystagmus [21,22,53], and—in some cases—fixational intrusions [21,23].

There are also larger—though less frequent—microsaccades during monocular fixation with amblyopic eyes [54] and similar microsaccadic impairments during binocular fixation of large targets [22,55]. The decreased microsaccade production scales with disease severity, with the fewest microsaccades found in patients with the most severe amblyopia [24]. Otero-Millan, Langston [56] demonstrated the same decreased frequency (but larger magnitude) of microsaccades in healthy controls when viewing stimuli that simulated amblyopia. These microsaccadic impairments are independent of increases to ocular drift [22], which exhibits increased velocity in amblyopia [23,57].

While many studies have found normal levels of fixation stability in the fellow eye of amblyopia patients [21,53,54,58,59], oculomotor abnormalities can occur in both the amblyopic and the fellow eye [22,60,61]. In fact, some of the abnormalities occur simultaneously in both eyes [62], suggesting the role of a central mechanism. In early infancy, during the emergence of stereopsis, decorrelated binocular signals in V1 can lead to downstream maldevelopment of extrastriate cortical areas MT/MST. These regions are responsible for maintaining gaze conjugacy [63]. Murray, Garg [64] suggested that fixational deficits may arise as a result, with cortical maldevelopment leading to the development of fusion maldevelopment nystagmus, which in turn prevents stable fixation.

These differences can be context-dependent: the atypical microsaccade rates in amblyopia do not translate into a difference in the typically observed decrease in microsaccade rate and subsequent rebound after the presentation of brief stimuli [25]. Moreover, Chen, Otero-Millan [24] found reduced frequencies of both small and large saccades in children with amblyopia during both fixation and visual search, but not during the exploration of a blank scene. Similarly, amblyopic fixation in the dark has been observed to be normal or close to normal [65,66]. However, results in this area are mixed: another study found abnormal fixation instability in amblyopia both when patients viewed blank scenes and when they viewed fixation targets [67]. Taken together, the evidence implies context-dependent changes to fixation instability in amblyopia. However, the specific contribution of task-relevant visual stimuli remains unresolved. Some stimulus effects also appear to be eye-specific, with one study reporting that the contrast of stimuli presented to the fellow eye primarily determined microsaccade amplitude of both eyes [68].

However, Raveendran, Bobier [69] induced retinal defocus by using convex lenses—simulating visual acuity deficits—and did not find a relationship between visual acuity deficits and fixation instability. Retinal defocus did not alter fixation stability for patients with amblyopia or for control participants, suggesting that visual acuity differences may not drive the fixation instability in amblyopia. As such, the role that visual acuity plays in fixation instability remains controversial. An alternative possibility is that increased drift may lead to lower visual acuity—as opposed to the other way around—by shifting retinal images to more eccentric locations [26,50]. During monocular fixation with a severely amblyopic eye, decreases in microsaccade production and increases in drift can also lead to perceptual fading for large regions of the visual field [26,50].

One study also concluded that fixation instability does not cause the motion perception deficits that can be seen in amblyopia [27].

Amblyopic patients with relatively substantial fixation instability, increases in drift, and relatively large microsaccades tend to have relatively poor visual acuity [21,23,58], as well as increased variability in visual acuity measurements [26,50]. In children, these deficiencies in visual acuity are linked to decreases in reading speed [70]. Fixation instability in these conditions is additionally associated with deficits in binocular vision and depth perception [26,71].

Some children with amblyopia, however, may improve their fixation stability as their visual systems continue to develop [72] or as treatment progresses [73].

Some researchers have attempted to improve fixation stability for the amblyopic eye—thus correctly aligning both eyes—by decreasing the contrast of images viewed by the fellow eye. This procedure did improve fixation stability, but only temporarily, as the amblyopic eye continued to drift away from alignment [59].

Fixational eye movement measures have also been proposed as a candidate treatment-monitoring outcome [26,71]. The approach is supported by findings that show some fixational eye movements do normalize after strabismus repair surgery, including reductions in both fixational saccade amplitude and drift velocity [28]. However, this was only true for patients without nystagmus, since for those patients, the frequency of fixational saccades (saccades produced during attempted fixation) and BCEA both did not improve after surgery [28]. Thus, while treatments can normalize fixational eye movements, this is condition-specific and has not been observed in all patients. Before clinical use, researchers must establish that these measures reliably track recovery and provide meaningful clinical information.

## 5. Cataracts

Patel, Wang [29] quantified fixation stability in children (age 4–13) with a history of dense unilateral cataract—after cataract extraction—and control participants. The participants in this study gazed binocularly at a stationary dot for 20 s. Relative to control participants, children with a history of the condition had less stable fixations in the eye that previously had a cataract and less stable vergence in the other eye. The authors suggested that, in children who had developed amblyopia because of cataracts, this instability might limit the recovery of their binocular vision.

In a study demonstrating that contrast sensitivity can be predicted from microsaccade dynamics, Denniss, Scholes [25] recorded eye movements from 9 patients with reduced vision from cataract, 10 patients with amblyopia, and 19 control participants with normal visual acuity. After the presentation of a brief—but visible—stimulus, microsaccade dynamics typically follow a pattern of a transient decrease followed by a “rebound” higher-than-baseline rate [74,75,76]. While some participants were found to not exhibit the expected decrease or rebound in microsaccade rates, no relationship was found between this heterogeneity across participants and their clinical group (control vs. cataract vs. amblyopia). The authors note that in future studies assessing microsaccade rate in clinical populations, methods should be used that are robust to this heterogeneity. Even control participants with normal visual acuity sometimes do not follow the classical pattern of a decrease then rebound in rate, and this should not be mistaken for clinical impairment.

## 6. Macular Disease and Central Scotoma

Macular disease causes various visual dysfunctions and also causes fixation instability [77,78]. This relationship can cause complications for eye tracking measurements in this clinical population. For example, Erbezci and Ozturk [30] attempted to evaluate the fixation stability of patients with age-related macular degeneration, but some patients (8 out of 72) were unable to fixate the target with both eyes. Moreover, the large lesions and/or substantially low visual acuity in these cases prevented microperimetry from being able to display the fixation area at all [30]—thus preventing fixation stability from being measured [see also [31]].

In patients where fixation stability could be calculated, Erbezci and Ozturk [30] found that visual acuity was positively correlated with fixation stability, consistent with other work investigating whether training patients to maintain stable fixations may improve their vision [79,80,81,82] (but see [32]). Biofeedback can be used to indicate to patients when their fixations are relatively stable or are in desired locations, but that training requires measurements of patients’ fixations. Unfortunately, difficulties in measuring the fixations of some patients (because their fixations are too unstable to readily measure using some techniques) may become a limiting factor in treatment for patients with such severe degeneration.

Fixation instability in macular disease may reflect increases in drift and a corresponding change in the amplitude and rate [33] or the velocity of (micro)saccades [33,83] (but see [84]). The less-affected eye can influence the more-affected eye, helping to normalize the movements to some degree. For example, fixation instability and microsaccade amplitude are more pronounced during monocular fixation with the eye most affected by maculopathy than during binocular fixation [34].

In macular disease, the increases in microsaccade amplitude are correlated with decreases in visual acuity [85], and the impaired fixation stability is strongly associated with decreased reading speeds in people with macular degeneration [35,86]. Déruaz, Matter [87] reported that a few patients were observed to intentionally shift their eyes quickly between different locations to improve their ability to identify peripherally presented letters for several seconds during a laboratory task, suggesting that such movements could prevent perceptual fading (sometimes called “Troxler’s effect”). However, while it is true that increased eye movements can prevent or reverse perceptual fading [5,8,88], individual letters are typically recognized in much less time than it takes for perceptual fading to occur (typically half a second or more). Therefore, a reduced level of fading might not fully account for the observed improvements to letter recognition.

Ağaoğlu and Chung [36] found that the abnormal fixational eye movements in macular disease also degrade the “spectral whitening” properties of fixational eye movements, in which the spectral content of retinal images is flattened. Spectral whitening removes redundancies in neural signals within the spatial frequency bands in which retinal ganglion cells are most sensitive [89,90]. The result of such degradation may be an impairment to the visual processing of fine spatial details.

In participants with normal vision, Otero-Millan, Langston [56] tested the effects of simulated scotomas meant to mimic the foveal vision loss in macular disease. Contrary to findings in patients with macular disease [33], microsaccade rate changed as a function of central vision loss: in the presence of the simulated scotomas, microsaccade production decreased and their magnitude increased. The authors attribute this difference to the lack of a stimulus that could serve as a “foveal anchor.” One speculative possibility is that a non-foveal preferred retinal locus could allow for more typical microsaccade production in the absence of foveal information. Prior studies have found that fixation stability improves over time as participants practice tasks with a simulated scotoma [91].

People with normal vision typically fixate their gaze to place visual areas of interest directly onto the fovea. However, for most patients where the fovea is no longer the region with the greatest visual acuity, they learn to use eccentric pseudo-foveae that serve as new reference points for the oculomotor system [92,93,94,95,96,97]. That is, patients learn to use “preferred retinal loci” (PRL), directing their gaze fixations to align visual stimuli onto one or more still-functional retinal regions outside of the fovea [95]. This is similar to the “averted vision” technique that amateur astronomers use to view faint stars, systematically looking off to the side of the stimulus they are trying to view in order to place it on the part of the retina where it will be the most visible [98].

Fixation stability is inversely related to the severity of macular disease, with fixations becoming less stable as the lesion size increases [99,100]. In patients where a very stable PRL has not been established, increased eye movements could reflect adaptive behaviors aimed at trying to locate a patch of retina that supports clear vision [101]. However, large lesions cause PRLs to be established further from central fixation, and the farther a gaze position is from central fixation, the less stable the resulting fixation [30,61,102,103]. Similarly, the farther the PRL is from the margins of the central lesion, the less stable the resulting fixation [30] (see also [61] for similar findings in amblyopia). PRLs can develop in any quadrant of the retina, with similar fixation stabilities across PRLs in different quadrants [104,105].

Patients often learn to adopt preferred retinal loci on their own. However, through microperimetry biofeedback and oculomotor exercises, patients can be trained to use new PRLs that focus visual stimuli on areas of optimal retinal sensitivity [81,106]. In fact, actively training patients to adopt more favorable PRLs can lead to increased fixation stability and to perceptual improvements [81,107,108]. The preferred retinal locus patients typically adopt is not necessarily the point of highest retinal sensitivity nor highest visual acuity—and is not necessarily the point that best facilitates the tasks (such as reading) that patients are trying to perform [81,107,108]. Biofeedback and eye movement training can lead to increased visual acuity.

When fixating well-trained preferred retinal loci, patients with macular disease have less stable fixations (with larger bivariate contour ellipse area values) than healthy controls fixating foveally [109]. Patients with gradual retinal damage have more time to adjust to a new preferred retinal locus. Consequently, adaptation may occur more readily than those with abrupt damage. One study compared the effects of gradual progression vs. abrupt progression with simulated lesions and found that fixations were less stable with abrupt scotomas than with gradual scotomas [110].

Training tasks that improve visual acuity or reading ability do not necessarily improve fixation stability [111,112]. Thus, eye movement training may be beneficial independently of other behavioral training approaches.

Measurement of fixation stability has sometimes been used to demonstrate the recovery of retinal function in clinical trials of potential treatments for macular disease. For example, one treatment being investigated for age-related macular degeneration involves the transplantation or translocation of healthy retinal pigment epithelium from a peripheral location (or a new epithelium patch engineered from embryonic stem cells) to the damaged location in the macula. Some studies found improved fixation stability that coincided with improvements in visual function after epithelial transplantation [31,113,114,115]. Microperimetry measurements in one study revealed that some patients were able to maintain stable central fixation years after the surgery [37], suggesting that this form of transplant may limit the progression of fixation instability in some patients.

Markowitz, Devenyi [116] found that photobiomodulation therapy (a form of low-level infrared light therapy) can improve fixation stability in some patients with dry age-related macular degeneration.

Vujosevic, Berton [117] found that fixation stability is unaffected by anti-vascular endothelial growth factor (anti-VEGF) treatment in macular edema. However, another study found that fixational eye movement abnormalities were reduced after treatment [38]. Because of these mixed results, it is currently unclear whether fixational eye movements should be explored as a candidate treatment-monitoring outcome for anti-VEGF treatment.

## 7. Retinal Detachment

The existing literature does not provide strong evidence for substantial long-term impairments in fixation stability or fixational eye movements. However, there have been relatively few studies in this area, and an absence of impairment has not been definitively established.

In a study of patients who had surgery (pars plana vitrectomy) to correct rhegmatogenous retinal detachment, Díaz-Barreda, Bartolomé-Sesé [39] did not find any difference in fixation stability between healthy controls and patients with rhegmatogenous retinal detachment (irrespective of whether or not the macula was involved in the detachment).

Dolar-Szczasny, Święch-Zubilewicz [40] found a small but significant decrease in fixation stability in patients where chronic central serous chorioretinopathy—a disorder in which fluid builds up under the retina and can cause retinal detachment—led to prolonged exposure to subretinal fluid, relative to age-matched controls.

## 8. Diabetic Retinopathy

Studies have mixed findings about the effects of diabetic retinopathy on fixation stability (as assessed by the fixation area of the bivariate contour ellipse area; BCEA). One study found no difference in fixation stability between normal participants and those with diabetic maculopathy [41], while others found that fixation is more unstable in diabetic retinopathy than in control groups [42,43]. Fixation stability does not reliably depend on the particular characteristics of any macular edema caused by diabetic retinopathy [42], although eyes with deposits of hard exudate exhibited less stable fixations. Poor fixation stability is more likely to be found in patients with relatively shorter durations of diabetes, retinopathy, or edema, possibly because patients who have experienced these visual deficits for a relatively short period of time have not yet developed a preferred retinal locus [118]. These findings should be interpreted as evidence about spatial dispersion under each study’s recording protocol rather than as evidence for a single underlying oculomotor mechanism. As such, no specific oculomotor signature of diabetic retinopathy has been established.

## 9. Glaucoma

**Figure 2:**
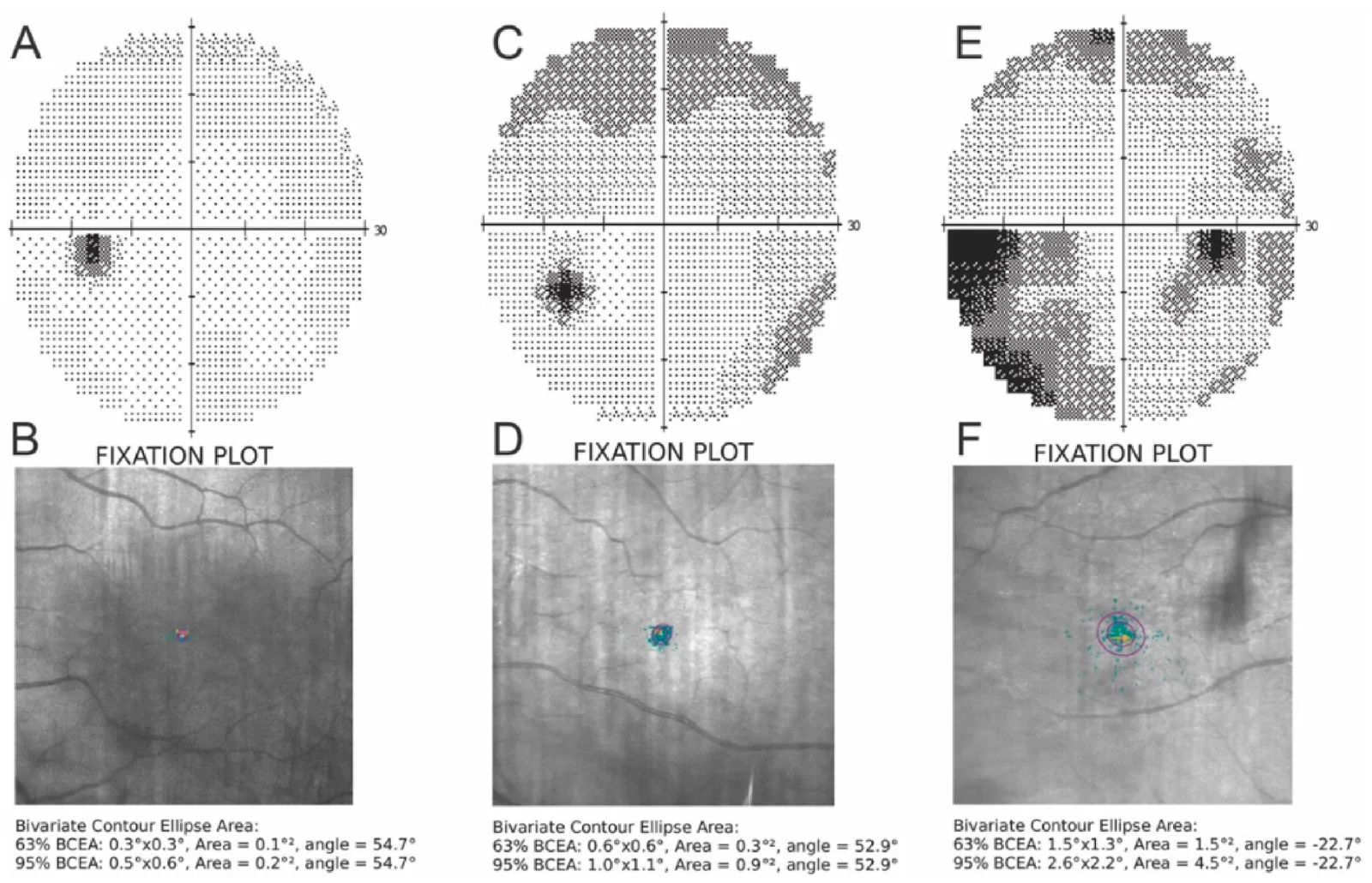
The visual fields and fixation patterns for different individuals across three stages of glaucoma: (**A**,**B**) a healthy eye, (**C**,**D**) an eye with mild primary open-angle glaucoma, and (**E**,**F**) an eye with moderate-to-severe primary open-angle glaucoma. The images demonstrate that fixation quality declines as the severity of glaucoma increases. Reproduced from Zabel, Zabel [44] without modification, under the terms of the Creative Commons BY-NC-ND 4.0 International License.

Glaucoma can impair fixation stability. For example, Zabel, Zabel [44] found that fixation stability in patients with primary open-angle glaucoma was negatively correlated with disease severity—see Figure 2. The authors proposed that fixation stability measures warrant further investigation and clinical validation as potential indicators for primary open-angle glaucoma.

Asfaw, Jones [45] examined patients with glaucoma who had asymmetric visual field loss between eyes. They found that patients had less-stable fixations when viewing images monocularly with their more affected eye than with their less affected eye.

Another study found decreases in fixation stability in glaucoma using a novel “Sequential Euclidean Distance” measure for capturing the spread of fixation positions [46]. When analyzing the same data with traditional measures of fixation instability (Bivariate Contour Ellipse Area or Mean Euclidean Distance), no difference was found. This provides a direct example of metric-dependent conclusions: the absence of an effect in BCEA does not necessarily establish normal fixation. There may be gaze differences that can only be detected through alternative approaches.

## 10. Limitations of the Current Literature and Future Directions

One difficulty in using fixational eye movement measures to assess health status is that subclinical impairments to fixational eye movements can be found in normal, control participants. For example, when measuring fixation stability, Stevenson and Roorda [119] mentioned that they found “a surprising” number of healthy individuals with subclinical jerk nystagmus. Similarly, square wave jerks are common in healthy observers [120]. Eye movement characteristics vary widely across individuals: for example, BCEA can expand dramatically with age even in the absence of pathology, due to routine lens stiffening or other minor factors [121]. These sources of normal variation limit the interpretability of isolated oculomotor measurements and underscore the need for established age-stratified, device-specific, and disease-specific reference ranges.

There is also substantial variation across different operational metrics. Microsaccades, drift, nystagmus, saccadic intrusions, BCEA, and general fixation instability all describe different oculomotor events or different levels of summary measurement. A condition may affect one or more of those metrics without necessarily impacting other fixational measures.

Because different diseases can produce partially distinct patterns of fixational eye movements, these measurements may eventually help differentiate between similar health conditions, although evidence supporting such applications remains limited. At present, using these motions as diagnostic markers remains premature. Diagnostic use will require comparative studies with appropriate clinical reference standards, prespecified classification thresholds, external validation, and explicit tests of robustness across disorders, disease severity, viewing conditions, recording systems, and patient populations. To date, much of the established research pertaining to fixational eye movements has centered around amblyopia, strabismus, and macular disease. Further work focused on fixational eye movements in other ophthalmic diseases must be conducted to establish a better foundational understanding of the ways in which ophthalmic diseases affect and alter oculomotor functions.

Future work should intentionally include diverse participant pools (in terms of race, ethnicity, sex, geographic region, and other factors) to ensure that findings can translate to global health contexts. Eye-tracking research often relies on the study of white, high-income populations. Moreover, eye tracking systems are often developed using primarily light-skinned participants and metrics suited to their ocular anatomy, resulting in reduced data quality for individuals with darker pigmentation or different eye physiology. For example, Nyström, Andersson [122] found that factors like eye color, eyelashes, and mascara significantly influence data quality. In addition, biomedical norms are disproportionately based on populations with access to diagnostic equipment, leaving large sections of the global community underrepresented in the foundational ophthalmic datasets on which future diagnostic tools will likely be based [123]. These gaps in the current, available data are exacerbated by the consistently small sample sizes in ophthalmic and eye movement studies, which typically lack the statistical power to assess intersectional effects on oculomotor behavior and disease progression. Larger, representative, multicenter datasets will therefore be necessary before fixational eye movement measures can be treated as generalizable biomarkers rather than promising laboratory correlates. Many clinicians and researchers are working to establish such datasets, as well as clinical standards for the use of oculomotor measures in clinical settings, which can help address some of those concerns [124].

Artificial intelligence-assisted analysis is likely to play an increasing role in this area, particularly for feature extraction, denoising, detection of atypical patterns, and integration of eye movement measures with retinal imaging. These uses should not be confused with clinical validation: algorithmic performance, including high classification accuracy within a dataset, cannot by itself establish clinical usefulness. AI-assisted classification will require external validation and representative samples. It will also require transparent reporting, comparison with existing clinical standards, and evidence that the models improve diagnosis, prognosis, treatment monitoring, or workflow efficiency.

Portable, virtual-reality-based, and wearable eye trackers may also expand the settings in which fixational measurements can be collected. In principle, these technologies can support lower-cost assessments and home-based detection of change between clinic visits. Their clinical promise depends on evidence that has not yet been established. Home and wearable monitoring will require substantial test–retest reliability assessments. Key concerns include calibration stability, variations in lighting and head-motion conditions, and patient adherence. Such monitoring will also need validation against established clinical systems. Without these safeguards, the addition of portable eye tracking may simply produce more unstable or unusable data.

A further priority is integration with optical coherence tomography, optical coherence tomography angiography, fundus photography, microperimetry, adaptive optics, visual field testing, and other multimodal retinal imaging approaches. Fixational measures are unlikely to replace these established methods in clinical practice. Their near-term value is more plausibly additive: they may help characterize how structural and functional abnormalities translate into oculomotor behaviors. Multimodal integration will require extensive testing and demonstrations to show that the measures add information beyond the existing tools alone.

For eye movement measures to be used effectively in clinical settings, the field should first generate normative ranges for the metrics, quantify within-session and between-session reliability, and test cross-device reproducibility. Subsequent studies should evaluate prospective and longitudinal validity, prespecify diagnostic thresholds, and determine whether changes in fixational measures predict disease progression or patient-centered outcomes. Sensitivity, specificity, predictive value, clinical utility, workflow impact, and cost-effectiveness will all need to be established. Until then, fixational eye movement measures should be considered promising research biomarkers and candidate monitoring outcomes, not validated diagnostic tests.

## 11. Discussion

Overall, substantial evidence has demonstrated that ophthalmic disease is often associated with modifications in fixation stability and fixational eye movements. That said, the measures discussed do not constitute a single interchangeable construct that we can universally apply when measuring oculomotor functions and differences across populations. Microsaccades, drift, nystagmus, saccadic intrusions, and BCEA describe different oculomotor events or different levels of summary measurement. Component measurements (microsaccade rate, drift velocity, etc.) can identify specific movement classes. Aggregate dispersion measures (such as BCEA), on the other hand, summarize spatial variability without identifying its source. To enable universally applicable diagnostic and monitoring tools, further work is needed to determine the reliability, specificity, cross-device comparability, and clinical utility of each metric under defined recording conditions and patient populations. Future studies must establish classification thresholds to identify and differentiate between benign impairment or variation and abnormal or deficient oculomotor behaviors. Subsequently, comparative studies will be needed to create and verify clinical reference standards. Additionally, as aforementioned, future work must establish greater diversity in population groups in terms of ethnicity, gender, geographic region, and other factors. In sum, fixational eye movement metrics currently represent a promising but still incompletely validated source of information about ophthalmic disease.

The most reproducible findings are of reduced fixation stability in amblyopia, strabismus, macular disease, and some glaucoma studies, which remain directionally consistent across multiple studies, disorders, and measurement contexts. Measures like drift velocity and microsaccade production are more mechanistically informative but are less explored. No specific change in metrics has been established yet as reliably disease-specific.

The strongest literature is currently concentrated in amblyopia and strabismus treatment studies and in macular disease studies involving PRL training, rehabilitation, transplantation, photobiomodulation, or anti-VEGF treatment. Even there, treatment responsiveness is measure-specific: some studies report improved fixation stability, drift, or saccadic amplitude, whereas microsaccade frequency, BCEA, or functional outcomes that do not always improve in parallel. This argues against using any single oculomotor measure as a universal clinical biomarker.

Before clinical implementation, there are significant remaining steps. Researchers must define the intended use of each metric; standardize acquisition, calibration, fixation targets, recording duration, preprocessing, and event detection. They must also establish age-, device-, and disease stage-specific normative ranges; quantify reliability; determine minimally meaningful change; establish reliability across participants and sites; and prospectively test the diagnostic, prognostic, and treatment-monitoring validity. Further work will need to demonstrate incremental value beyond established measures and to evaluate feasibility, workflow burden, equity, and cost. Until those steps are completed and findings are clinically validated, the defensible clinical position is narrow: fixational measures are promising exploratory correlates of disease (and in some cases, visual dysfunction) but are not yet established as stand-alone diagnostic tests or disease-specific signatures.

## Figures and Tables

**Table 1:** Characteristics of included studies reporting fixational eye movement alterations in ophthalmic disease.

Ophthalmic Disorder	First Author, Year	Study Design	Sample Characteristics and Sample Size	Monocular/Binocular Recording	Recording System	Fixational Measures	Principal Finding
Myopia and other refractive disorders	Yang et al., 2022 [15]	Cross-sectional observational study	Young adults with different degrees of myopia. N = 91	Binocular	Nidec ARK-1 and Tobii Eye Tracker 4C	BCEA	Greater axial length was associated with less increased 95% BCEA
Myopia and other refractive disorders	Zhu et al., 2017 [16]	Cross-sectional observational study	Highly myopic eyes undergoing biometry measurements and controls	Not specified	Macular Integrity Assessment (MAIA) microperimetry system	BCEA	Refractive errors and 63% BCEA were correlated with axial length
Myopia and other refractive disorders	Zhu et al., 2016 [17]	Cross-sectional observational study	98 patients with myopia and 42 controls, after cataract surgery. N = 140	Monocular	Macular Integrity Assessment (MAIA) microperimetry system	BCEA	Highly myopic cataract group had greater hyperopic refractive errors. BCEA (at both 63% and 95% contour levels) was associated with refractive error after cataract surgery
Myopia and other refractive disorders	Ghasia and Shaikh, 2015 [18]	Experimental simulation study	Participants with corrected and uncorrected myopic refractive error. N = 10	Binocular	High-resolution video oculography	Microsaccade amplitude, velocity, and rate	Uncorrected myopic refractive error increased microsaccade amplitude without altering microsaccade velocity or rate
Myopia and other refractive disorders	Zhu et al., 2019 [19]	Cross-sectional observational study	500 Myopic patients and 50 control patients aged 30+ who were scheduled for cataract surgery. N = 550	Binocular	Macular Integrity Assessment (MAIA) microperimetry system	BCEA; fixation-ellipse orientation	Axial length was positively associated with superiorly shifted BCEA ellipses (at both the 63% and 95% contour levels)
Amblyopia and strabismus	Ghasia et al., 2017 [20]	Cross-sectional observational study	13 patients with strabismus and 16 controls. N = 29	Monocular	EyeLink 1000 eye tracker	Drift velocity; fixational saccades; conjugacy; intersaccadic drifts	Strabismus was associated with increased 68% BCEA, drift, and more disconjugate fixational saccades
Amblyopia and strabismus	Subramanian et al., 2013 [21]	Cross-sectional observational study	31 children (5–17 years old) with strabismus, 29 with anisometropia, and 29 children with both conditions. N = 89	Monocular	Nidek MP-1 microperimeter	BCEA	Reduced fixation stability was observed in amblyopia (assessed with BCEA at 95% contour level); fellow-eye stability was often normal
Amblyopia and strabismus	Shaikh et al., 2016 [22]	Cross-sectional observational study	36 patients with amblyopia, 11 controls. N = 47	Binocular	High-resolution video-oculography	Microsaccade production; drift velocity	Amblyopia was associated with reduced or altered microsaccade production and increased drift velocity
Amblyopia and strabismus	Kang et al., 2019 [23]	Cross-sectional observational study	44 patients with amblyopia and 20 controls. N = 64	Monocular and binocular	EyeLink 1000 eye tracker	Fast and slow fixational eye movement waveforms; fixational saccade amplitude; inter-saccadic drift; BCEA; drift velocity; disconjugacy	Patients with amblyopia had increased BCEA (68% contour level), fixational saccade amplitude, drift variance, drift velocity, and disconjugacy versus controls
Amblyopia and strabismus	Chen et al., 2018 [24]	Cross-sectional observational study	21 children with amblyopia and 10 controls. N = 31	Monocular	Infrared video-oculography	Microsaccade amplitude; microsaccade frequency; intersaccadic drifts	Children with amblyopia made fewer small and large saccades during fixation and visual search, but not during blank-scene exploration. Across all tasks, amblyopic participants increased intersaccadic drifts
Amblyopia and stra-bismus; Cataracts	Denniss et al., 2018 [25]	Cross-sectional observational study	9 patients with reduced vision from cataract, 10 patients with amblyopia, and 19 controls. N = 38	Binocular	EyeLink 1000 eye tracker	Microsaccade rate	Heterogeneity in microsaccade suppression and rebound was not specific to cataract or amblyopia
Amblyopia and strabismus	Ciuffreda et al., 1979 [26]	Cross-sectional observational study	4 patients with amblyopia, 13 patients with amblyopia and strabismus, and 5 patients with strabismus. N = 22.	Monocular and binocular	Photoelectric method	Drift velocity; saccadic intrusions; nystagmus	Fixational eye movements were abnormal in amblyopia and strabismus. Increased drift was related to the presence of amblyopia, while saccadic intrusions and nystagmus were related to strabismus
Amblyopia and strabismus	Meier et al., 2025 [27]	Cross-sectional observational study	Patients with amblyopia. N = 13	Monocular	EyeLink 1000 eye tracker	BCEA	Poor fixation stability (assessed with BCEA at 95% contour level) did not account for motion perception deficits in amblyopia
Amblyopia and strabismus	Martin et al., 2021 [28]	Pre-post treatment study	12 patients with fusion maldevelopment nystagmus and 9 without nystagmus undergoing strabismus repair N = 21	Binocular	EyeLink 1000 eye tracker	Fixational saccade amplitude; inter-saccadic drifts; drift velocity; microsaccade frequency; BCEA	Fixational saccade amplitude and intersaccadic drift velocity normalized after strabismus repair in patients without nystagmus; fixational saccade frequency and BCEA (95% contour level) did not improve in patients with nystagmus
Cataracts	Patel et al., 2021 [29]	Cross-sectional observational study	17 children (4–13 years old) with a history of dense unilateral cataract, and 46 similar-aged controls. N = 63	Binocular	EyeLink 1000 eye tracker	BCEA (contour level not indicated); vergence stability	Children with prior unilateral cataract showed less stable fixation in the previously affected eye and less stable vergence in the other eye
Macular disease and central scotoma	Erbezci and Ozturk, 2018 [30]	Cross-sectional observational study	Patients with macular degeneration. N = 72	Monocular	Scanning laser ophthalmoscope (SLO) microperimeter	Fixation stability (as assessed by distance between the most distant gaze positions on the fundus image); PRL location	Visual acuity was positively correlated with fixation stability; severe disease sometimes prevented fixation-area display
Macular disease and central scotoma	Rizzo et al., 2020 [31]	Clinical Intervention (Treatment-monitoring study)	Patients with age-related macular degeneration, 5 affected by geographic atrophy AMD, and 6 affected by CNV, who underwent a pars plana viterctomy. N = 11	Monocular	Microperimetry	BCEA	Reduced 68% BCEA (improved fixation stability) coincided with functional improvement after treatment
Macular disease and central scotoma	Rubin et al., 2024 [32]	Randomized clinical rehabilitation/intervention trial	Patients with age-related macular disease. N = 200	Monocular	Nidek MP-1 microperimeter	BCEA	Eccentric viewing training did not provide consistent support for improved fixation stability (assessed by 68% BCEA)
Macular disease and central scotoma	Kumar and Chung, 2014 [33]	Cross-sectional observational study	16 participants with macular disease, 14 controls. N = 30	Monocular	Rodenstock scanning laser ophthalmoscope (SLO) microperimeter	BCEA; Drift velocity; microsaccade amplitude and rate	Fixation instability (assessed by 65% BCEA) may reflect increased drift and altered microsaccadic dynamics
Diabetic retinopathy	Jakobsen et al., 2017 [34]	Cross-sectional observational study	Patients with center-involving diabetic macular edema. N = 29	Monocular recording (monocular and binocular stimuli)	iView X TM HighSpeed eye tracker	Area of fixation; fixational saccade amplitude and frequency	Binocular fixation reduced fixational eye movements in the worse eye
Macular disease and central scotoma	Crossland et al., 2004 [35]	Cross-sectional observational study	Patients with juvenile macular disease, who had developed a scotoma in their better eye. N = 25	Not specified	Scanning laser ophthalmoscope (SLO) microperimeter and an infra-red gaze tracker	BCEA	Impaired fixation stability (BCEA at 68.2%, 95.4%, and 99.6%) was associated with decreased reading speed
Macular disease and central scotoma	Ağaoğlu and Chung, 2020 [36]	Cross-sectional observational study	15 participants with macular disease, 14 older adults with normal vision, and 7 young adults with normal vision. N = 36	Monocular	Rodenstock Model 101 Scanning Laser Ophthalmoscope (SLO 101) microperimeter	Drift amplitude; microsaccade amplitude	Abnormal fixational eye movements (assessed by 68% isoline area and ocular drift characteristics) degraded spectral whitening of retinal-image motion
Macular disease and central scotoma	Parolini et al., 2020 [37]	Retrospective observational study	Patients that underwent retinal pigment epithelium –choroid patch surgery for exudative and atrophic macular degeneration by the same surgeon. N = 84	Binocular	Macular Integrity Assessment (MAIA) microperimetry system	Fixation stability	Years after surgery, some patients maintained stable central fixation (as measured by whether or not >75% of fixation points were within 1 or 2 degrees of visual angle of the PRL)
Diabetic retinopathy	Jakobsen et al., 2018 [38]	Longitudinal observational study (Treatment-monitoring study)	Patients with diabetic macular edema receiving anti-vascular endothelial growth factor treatment. N = 29	Only monocular data were used	iView X TM HighSpeed eye tracker	BCEA; fixational saccade frequency	68% BCEA covaried with short-term changes in visual acuity after anti-VEGF treatment
Retinal detachment	Díaz-Barreda et al., 2021 [39]	Cross-sectional observational study	38 patients with rhegmatogenous retinal detachment (19 macula-on and 19 macula-off) and 113 controls. N = 151	Monocular	Macular Integrity Assessment (MAIA) microperimetry system	BCEA	No difference in 63% or 95% BCEA was found between controls and patients after retinal detachment surgery
Retinal detachment	Dolar-Szczasny et al., 2018 [40]	Cross-sectional observational study	Patients with chronic central serous chorioretinopathy. N = 15	Monocular	Optopol SOCT Copernicus HR spectral domain optical coherence tomography (SD-OCT), Heidelberg Retina Angiograph 2 (HRA2) Scanning Laser Ophthalmoscope, and Macular Integrity Assessment (MAIA) microperimetry system	Percent of fixations near the fixation target	Prolonged exposure to subretinal fluid was associated with a small but significant decrease in fixation stability (as measured by whether or not >75% of fixation points were within 1 or 2 degrees of visual angle) relative to controls
Diabetic retinopathy	Dunbar et al., 2010 [41]	Cross-sectional observational study	21 patients with diabetic maculopathy and 16 controls. N = 37	Monocular	Nidek MP-1 microperimeter and Rodenstock scanning laser ophthalmoscope	BCEA	No difference in 68% BCEA was found between participants with diabetic maculopathy and controls
Diabetic retinopathy	Vujosevic et al., 2008 [42]	Cross-sectional observational study	Patients with diabetic macular edema. N = 98	Binocular	Nidek MP-1 microperimeter	Fixation stability (as measured by whether or not >75% of fixation points were within 2- or 4-degrees radius of the fixation points)	Fixation stability did not reliably depend on the specific characteristics of macular edema.
Diabetic retinopathy	Carpineto et al., 2007 [43]	Cross-sectional observational study	Type 2 diabetic patients with diffuse macular edema. N = 84	Monocular	Nidek MP-1 microperimeter	Fixation stability (as measured by whether or not >75% of fixation points were within 2 degrees radius of the fixation points)	Fixation was more unstable in diabetic macular edema than in control groups
Glaucoma	Zabel et al., 2022 [44]	Cross-sectional observational study	32 patients with primary open-angle glaucoma and 16 controls. N = 48 eyes	Monocular	Macular Integrity Assessment (MAIA) microperimetry system	BCEA	95% BCEA was negatively correlated with disease severity.
Glaucoma	Asfaw et al., 2018 [45]	Cross-sectional observational study	Patients with glaucoma and asymmetric visual-field loss. N = 15	Monocular between-eye comparisons	EyeLink 1000 eye tracker	BCEA	Fixations were less stable when patients viewed images with the more affected eye than with the less affected eye (as assessed by 95% BCEA).
Glaucoma	Montesano et al., 2018 [46]	Cross-sectional observational study	120 patients with glaucoma and 200 controls. N = 320	Monocular	iCare Compass microperimeter	BCEA	Sequential Euclidean Distance detected reduced fixation stability in glaucoma, whereas traditional 95% BCEA and Mean Euclidean Distance did not.

## Data Availability

No new data were created or analyzed in this study. Data sharing is not applicable to this article.

## Abbreviations

The following abbreviations are used in this manuscript:

AL	Axial Length
BCEA	Bivariate Contour Ellipse Area
MT	Middle Temporal area
MST	Medial Superior Temporal area
PRL	Preferred Retinal Locus
